# Pituitary Adenylate Cyclase Activating Peptide and Post-traumatic Stress Disorder: From Bench to Bedside

**DOI:** 10.3389/fpsyt.2022.861606

**Published:** 2022-07-05

**Authors:** Manessa Riser, Seth Davin Norrholm

**Affiliations:** Department of Psychiatry and Behavioral Neurosciences, Neuroscience Center for Anxiety, Stress, and Trauma, Wayne State University School of Medicine, Detroit, MI, United States

**Keywords:** trauma, peptides, stress, translational neuroscience, HPA—hypothalamic-pituitary-adrenal, fear and anxiety, estrogen

## Abstract

Pituitary adenylate cyclase-activating polypeptide (PACAP) is a neuropeptide with isoforms consisting of either 27 or 38 amino acids. PACAP is encoded by the adenylate cyclase activating peptide gene, *ADCYAP1*, in humans and the highly conserved corresponding rodent gene, *Adcyap1*. PACAP is known to regulate cellular stress responses in mammals. PACAP is robustly expressed in both central nervous system (CNS) and peripheral tissues. The activity of PACAP and its selective receptor, PAC1-R, has been characterized within the hypothalamic-pituitary-adrenal (HPA) axis and autonomic division of the peripheral nervous system, two critical neurobiological systems mediating responses to stressors and threats. Findings from previous translational, empirical studies imply PACAP regulation in autonomic functions and high expressions of PACAP and PAC1 receptor in hypothalamic and limbic structures, underlying its critical role in learning and memory, as well as emotion and fear processing. The current review summarizes recent findings supporting a role of PACAP/PAC1-R regulation in key brain areas that mediate adaptive behavioral and neurobiological responses to environmental stressors and maladaptive reactions to stress including the development of fear and anxiety disorders.

## Introduction

### Pituitary Adenylate Cyclase Activating Polypeptide

Pituitary adenylate cyclase-activating polypeptide (PACAP) is a neuropeptide biologically existing as two variants, consisting of either 27 or 38 amino acids residues, named PACAP27 and PACAP38, respectively (1). PACAP38 is 10–100-fold more plentiful in central and peripheral nervous tissue than PACAP27 (2). It was isolated from ovine hypothalamic tissues over three decades ago and named based on its ability to stimulate adenylate cyclase activity (and, in turn, cAMP) in rat pituitary cells (3–5). PACAP belongs to the vasoactive intestinal peptide (VIP)-secretin-glucagon family of bioactive peptides (6) with its short 27-amino acid form sharing 70% homology with VIP (7). PACAP functions in a broad array, ranging from development to metabolism to cell signaling mechanisms and shows remarkable evolutionary conservation across time and species (7–9). Expressed both centrally and peripherally in the nervous system, it is known to regulate physiological and psychological stress responses at the cellular level across species (10).

**FIGURE 1:**
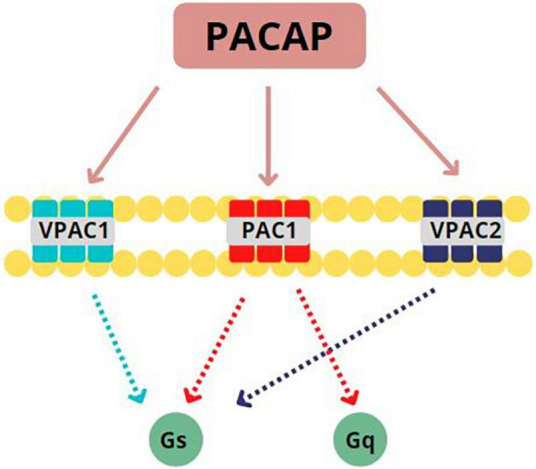
Pituitary Adenylate Cyclase Activating Peptide (PACAP) binds to three G-protein coupled receptors (GPCRs): PACAP binds to three GPCRs: VPAC1, VPAC2, and PAC1. PAC1 is specifically selective for PACAP27 and PACAP38, binding with high affinity. Once bound to its associated GPCRs, PACAP activates Gs or Gq subunits. This activation leads to the initiation of adenylate cyclase activity to stimulate the conversion of ATP to cAMP or the initiation of phospholipase C activity to stimulate protein kinase C. Adapted from Rubio-Beltrán et al. (65).

PACAP binds to three G-protein coupled receptors (GPCRs): VPAC1, VPAC2, and PAC1 (see Figure 1). Only the PAC1 receptor is selective for both PACAP27 and PACAP38 with high affinity (11) and more than 20 isoforms of this subtype have been identified as of this writing (12). PACAP can presynaptically and postsynaptically activate PAC1 receptors, behaving as both a typical synaptic transmitter as well as a modulator of neuronal activity (13). Once bound to its associated GPCRs, PACAP activates Gs or Gq subunits to initiate either (1) adenylate cyclase enzymatic activity which, in turn, stimulates conversion of ATP to cAMP which then phosphorylates protein kinase A (PKA) to produce secondary downstream effects (14–16) or (2) phospholipase C (PLC) activity to stimulate protein kinase C (PKC) or 1,4,5 inositol triphosphate (IP3) second messenger cascades (17–19). Based on these postsynaptic actions, PACAP is well positioned in mammalian CNS to influence numerous processes related to neurodevelopment, neuroprotection, and neuromodulation (16, 20).

Based on a myriad of studies, PACAP and its selective receptor, PAC1-R, are associated with the regulation of the hypothalamic pituitary adrenal (HPA) axis, which is the principle hormonal stress response system in mammals. The HPA axis primarily functions to induce the production and release of hormones through the modulation of corticotrophin releasing hormone (CRH, also termed corticotropin releasing factor, CRF). CRH is essential to regulating physiological responses to stress exposure. As such, CRH expression is highest in brain regions that respond to stressors such as the brainstem locus coeruleus (LC), bed nucleus of the stria terminalis (BNST), and the central nucleus of the amygdala (CeA). Overactivity of CRH has been implicated in the pathophysiology of the human psychiatric condition post-traumatic stress disorder (PTSD) (21). The accumulating body of literature regarding PACAP indicates similar regulatory stress response activity that may occur upstream to CRH-mediated processes.

Although similarly implicated in mammalian stress responding as other hormones (e.g., adrenocorticotropic hormone, ACTH), PACAP is unique in a number of key areas. For example, PACAP possesses a basic charge, circulates within ceruloplasmin complexes known to transfer positively charged ions like copper (22), has no definitive target organ or tissue, circulates in markedly lower plasma levels than other peptides (American College of Physicians, Normal Laboratory Values),^1^ and appears to act as a “paracrine operator” more so than a systemic “master regulator” (23). Its widespread expression and function are exemplified by the role of this peptide in CO_2_ chemosensitivity (24), neonatal stress responses in rodent maternal separation paradigms (25) and human sudden infant death syndrome (SIDS) (24), as well as in acute, autonomic, and hypoxic stress responses (26).

PACAP and PAC1 receptors are richly and widely distributed throughout the central nervous system (CNS) and peripheral tissues. PACAP and its receptor, PAC1-R, have been identified in the HPA axis and autonomic stress systems. PACAP is expressed in nuclei of the brainstem and hypothalamus, amygdala, posterior pituitary, and thalamic regions of the brain (27). In peripheral organs, PACAP is expressed in the exocrine and endocrinal glands as well as in the gonads. PAC1-Rs are broadly expressed in cortex, hippocampus, brain stem, olfactory bulb, cerebellum, and hypothalamus (28, 29).

The highest concentration of PACAP38 in the CNS is observed in the paraventricular nuclei (PVN) of the hypothalamus (30). PACAP-positive terminals can synapse with PVN CRH neurons to trigger CRH production and secretion. The high expression of PACAP in hypothalamic and limbic regions of the brain support an integral role in learning and memory, including emotion regulation and fear processing. The integration of empirical research using animal models and human samples implicate PACAPergic systems in stress neurobiology, specifically how persistent changes in signaling and expression may result in stressor- and fear-related psychopathologies (30–32). With regard to learning and memory, PACAP possesses the ability to activate signaling cascades that are closely tied to memory formation (33, 34). Specifically, adenylate cyclase (the enzymatic portion of PACAP’s namesake) signaling is critical to the cellular basis of memory consolidation through the mediation of cAMP-response-element-binding (CREB) protein on gene transcription (35). It appears as though it is through such action that PACAP mediates fear learning during highly salient, unpleasant experiences and their subsequent development into adverse psychopathology (36).

Clinically speaking, PACAP has been implicated in well over 40 medical and psychiatric disorders to date spanning migraine to stroke to PTSD (16, 23), however, the focus of the present review will be on PTSD and its closely related co-morbidities.

## Post-Traumatic Stress Disorder

Posttraumatic stress disorder (PTSD) is a severe psychiatric disorder that occurs in people who have witnessed or experienced a psychologically distressing or traumatic event (e.g., motor vehicle accident; military deployment to combat theater; violent physical or sexual assault). PTSD is characterized according to sign and symptom phenotypes: re-experiencing/intrusion (e.g., flashbacks), avoidance/negative alterations in mood or cognition (e.g., distorted views of self or world), and alterations in arousal and reactivity (e.g., irritability, hypervigilance, disturbed sleep). PTSD has a prevalence of 8.3% according to a 2013 study conducted by Kilpatrick (37) and colleagues comparing DSM-IV and DSM-5 criteria of trauma exposure and PTSD in a national sample of adults in the US. Clinical PTSD is typified by a change in reactivity to stressful experiences, with a strong prevalence in women (36). In general, women are twice as likely as men to develop PTSD (38). The sex bias in stressor- and fear-related disorders can be attributed not only to sex differences in social, societal, and psychological factors, but to sex-dependent divergence in the interactions of sex hormones within limbic neurocircuitry (13).

## Moving From Bench to Bedside: Pituitary Adenylate Cyclase-Activating Polypeptide and Responses to Stressors or Threat

**FIGURE 2:**
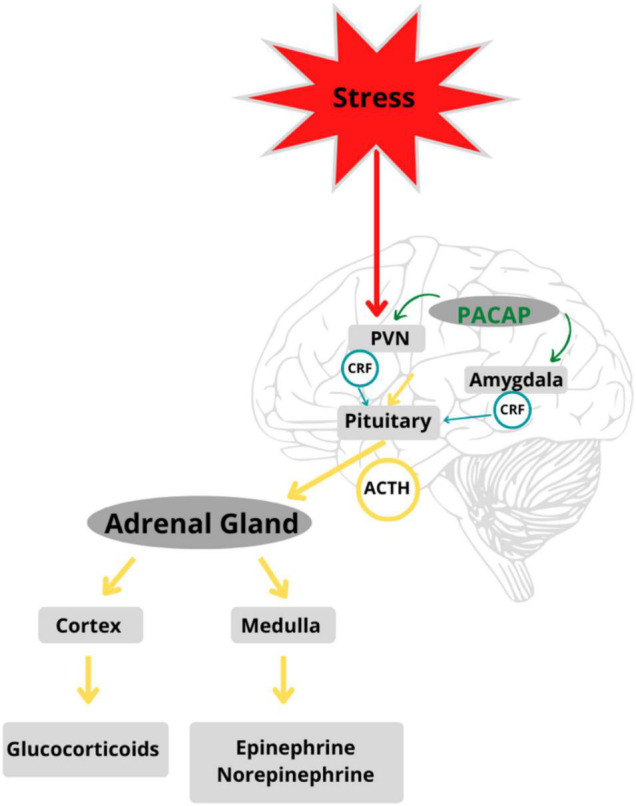
PACAP is a regulator of stress circuits in the HPA axis: PACAP modulates feedback between glands in the HPA axis mediating stress response in the body. Adapted from Stroth et al. (56).

When an event or stimulus is perceived, consciously or unconsciously, as stressful, there is feedback between glands along the HPA axis that stimulate the release of stress hormones and the subsequent recruitment of neural and somatic response systems (39–42). Different types of stressful stimuli (i.e., physical, emotional, or metabolic) activate specific neurocircuitry in the stress-mediating axes. The degree of activation of the HPA axis, adrenomedullary hormonal system, and sympathetic nervous system is reflected in nerve firing patterns in cells that release stress-mediating hormones such as ACTH, adrenaline, and noradrenaline (43). Stress response circuits involve synchronized activation of the HPA and neuroendocrine systems (44). All stress responses are centrally integrated in the PVN of the hypothalamus, which functions as a coordinating center (45), peripherally affected by the adrenomedullary neuroendocrine systems. The hormonal cascades initiated by stress responses led to further investigation of PACAP and its putative role in the stress axis (see Figure 2).

Several lines of evidence derived from rodent studies aided in the revelation of PACAPergic activity in mammalian stress responding [e.g., (46)]. PACAP-38 was found to induce catecholamine release from the adrenal medulla via action of sympatho-adrenal projections as part of threat-related activation of the autonomic nervous system (47, 48). In short, PACAP is expressed in preganglionic sympathetic neurons within the intermediolateral cell column of the thoracolumbar spinal cord. As such, PACAPergic innervation of sympathetic ganglia and the dense expression of PAC1 receptors on postganglionic sympathetic neurons provides a mechanism for PACAP to robustly stimulate stress-associated catecholamine release [for review see (21)].

Further, significant PACAP-38 and PAC1-R expression occurs in several limbic regions mediating stress responses including the extended amygdala (and bed nucleus of the stria terminalis, BNST), hippocampus, and medial prefrontal cortex (mPFC) (49–53). Further, PACAP-38 was not only found to be expressed in projections onto CRH neurons in the hypothalamic PVN (53) but the peptide could also increase CRH expression (54) and mediate prolonged stress responses through modulation of CRH release (43).

In addition to the molecular evidence discussed in the preceding section, there are several behavioral studies of note related to PACAP and its increasingly understood role in rodent stress responses. For example, Legradi et al. (55) assessed the modulatory role of PACAP in fear responses through action at the central nucleus of the amygdala (CeA) in male Sprague-Dawley rats. In this study, the experimental group was microinfused with 50–100 pmol of PACAP via intra-CeA cannulae, then shock-stressed. Results demonstrated that intra-CeA PACAP injections increased the likelihood of fear and anxiety related behaviors (e.g., withdrawal and freezing) in shock-stressed rats compared to controls. Later, in 2010 and 2011, Stroth and Eiden identified PACAP as a major contributor to the process of stimulus-secretion-synthesis coupling. Male mice (PACAP−/− and PACAP +/+) were subjected to continuous restraint for varying lengths (1, 3, or 6 h). Their results further implicated the involvement of PACAP signaling in sustained corticosterone secretion, synthesis of CRH in the hypothalamus, and production of adrenaline in the adrenal medulla (43, 56). Compared to wildtypes, the concentrations of corticosterone in PACAP deficient mice were reduced by more than 60% (56). This reduction was greater in mice that experienced longer durations of restraint.

Tsukiyama et al. (57) also explored the association between PACAP and emotional stress-induced corticosterone responses in PACAP-deficient mice by examining consequences of four contrasting types of acute stressors: open-field exposure (emotional), cold exposure (metabolic), ether inhalation (physical), and restraint stress (physical). The latter group demonstrated reduced corticosterone release under two of the four conditions: open-field exposure and restraint stress (57).

Relevant to maladaptive responses to stressors and threat, PACAP has also been implicated in the development, expression, and maintenance of conditioned fear; an associative learning process believed to underlie many fear- and anxiety-related disorders (58). Prior work has shown that PACAP-38 has profound short- and long-term effects on the consolidation of learned fear through activity within the BNST, amygdala, and PFC (59–61), presumably at PAC1-Rs (62).

Transgenic PACAP- or PAC1 receptor-deficient rodents have been generated across multiple studies to better understand the physiological and psychological functions of PACAP in responses to stressors and threats. In a series of experiments using classical fear conditioning, Ressler et al. (63) showed an association between fear consolidation and PAC1 receptor genomics when comparing peak freezing behaviors. mRNA expression of *Adcyap1r1* was significantly increased in the amygdala during consolidation of fear. An analogous trend was observed in mPFC. Evidence from these rodent models support the connection between PACAP/PAC1 receptor signaling, activity in the amygdala and BNST, and PTSD symptomatology (41).

It has been demonstrated that PACAP is clearly implicated in fear learning processes and that these processes recruit limbic brain regions as part of the expression and catabolism of learned fear. A brain region that encapsulates the interplay between this neuropeptide and fear learning is the hippocampus and, more specifically, the dentate gyrus (DG) hippocampal subfield (11). For example, the PACAP-selective receptor, PAC1, is highly expressed in the granule cell layer of DG specifically (28, 64) and, as such, well positioned to influence fear behaviors mediated by hippocampal function.

## Pituitary Adenylate Cyclase-Activating Polypeptide: At the Bedside

As described earlier, more than 20 PACAP receptor variants have been reported in vertebrates with differing ligand selectivity and second messenger cascade activation. As such, this peptide is believed to have tissue-specific expression and signaling with tissue-specific adaptive and maladaptive responses (23). For example, PACAP signaling has been implicated in the molecular underpinnings mediating, and the potential treatment of, migraine (65, 66), ischemia/stroke (16), Alzheimer’s disease (67), and PTSD (63). Evidence for a wide-ranging role for PACAP in several neurological and psychological disorders stems from the expression of this peptide along several nodes of the pathways mediating pain, emotion, fear, anxiety, and stress including sensory dorsal root and trigeminal ganglion neurons and lateral amygdala (16, 68).

### Pituitary Adenylate Cyclase-Activating Polypeptide and Human Responses to Stressors or Threat

Complementary to rodent models, recent research suggests that changes in PACAP expression and signaling may give rise to psychopathologies related to stress exposure and fear behaviors in humans. PACAP signaling has been associated with numerous psychiatric disorders and single nucleotide polymorphisms (SNPs) along the PAC1 receptor gene, *ADCYAP1R1*. Even further, there are differential interactions between stress, PACAP, and hormonal responsiveness when comparing males and females (13, 63). This distinctive sexually dimorphic hormonal regulation may impact behaviors related to anxiety and fear, while driving the sex-related disparities in the prevalence of psychiatric disorders. One of the most essential hormones to consider is estrogen. Estrogen and estrogen receptors have been shown to exhibit modulatory control over several neurotransmitter systems, while simultaneously influencing activity of the limbic and HPA pathways and behavioral stress responses (13). Interestingly, findings suggest that PACAP may be a mechanistic pathway through which estrogen can mitigate the effects of chronic stress in females.

High levels of PACAP38 were specifically associated with PTSD symptomatology in African American females (with PTSD diagnosis) according to a 2011 study by Ressler and colleagues. When stratified according to three phenotypes of PTSD symptoms (intrusions, avoidance, and hyperarousal), significant associations were maintained, with the most robust association being observed in the hyperarousal subscales. Additionally, acoustic startle measures indicated that females with high PACAP levels had enhanced startle responses to both fear and safety cues, suggesting an impairment in fear discrimination. In an aim to further understand these findings, Mercer et al. (69) found a negative relationship between PTSD symptomatology and the expression of the PACAP gene in blood mRNA of human subjects. Their research demonstrated contrasting results, when compared to the findings of Ressler et al. (63) in which the severity of PTSD symptoms was actually associated with lower expressions of PACAP (69).

Investigators have examined 44 SNPs in the PACAP/PAC1-R genetic coding (*ADCYAP1*/*ADCYAP1R1*), identifying only one SNP, rs2267735, that was significantly associated with females in PTSD cohorts. The risk genotype, *rs2267735 with homogenous “CC” allele*, increased the risk of PTSD in a sample made up of about 40% of African American females, evidenced by the lack of association to other severe psychiatric disorders (2, 70). However, associations between PACAP expression and PTSD remains controversial. A study conducted by Almli et al. (37), found an interaction between genotype and trauma, but failed to replicate the findings from previous studies that found a main effect of *ADCYAP1R1* on PTSD risks in females.

## Future Implications

The severity and onset of many stressor- and fear-related psychopathologies are associated with the type, degree, duration, and chronicity of stress exposure. Further, abnormal expression and functioning of PACAP and other hormones have been implicated in these disorders (13). Understanding the genetic and environmental interactions of PACAP (*ADCYAP1*) and PAC1-R (*ADCYAP1R1*) on neural plasticity is essential for comprehending the mechanistic pathways that underlie stress and fear related pathologies. These data indicate that trauma “dose” differentially interacts with PACAP/PAC1-R genetic risk factors and may reflect conditional physiological and psychological responses to traumatic experiences. Advancing the development of treatment and preventative methods for traumatized populations requires well-defined risk criteria for intervention (37). Furthermore, understanding the key components illustrated in this review builds the foundation for therapeutic approaches to fear- and stressor-related psychopathologies.

## Author Contributions

MR and SN wrote each draft of the manuscript and its individual sections. Both authors contributed to manuscript development, read, and approved the submitted version.

## Conflict of Interest

The authors declare that the research was conducted in the absence of any commercial or financial relationships that could be construed as a potential conflict of interest.

## Publisher’s Note

All claims expressed in this article are solely those of the authors and do not necessarily represent those of their affiliated organizations, or those of the publisher, the editors and the reviewers. Any product that may be evaluated in this article, or claim that may be made by its manufacturer, is not guaranteed or endorsed by the publisher.

## Footnotes

1
www.acponline.org
